# Supplementary material for: Evaluation of the Harvest Dates for Three Major Cultivars of Blue Honeysuckle (Lonicera caerulea L.) in China
Source: Plants (Basel). 2023 Nov 2;12(21):3758. doi: 10.3390/plants12213758 (PMC10649999; doi:10.3390/plants12213758)
Supplement: Supplementary file 1 [file plants-12-03758-s001.zip › plants-2595999-supplementary.pdf]

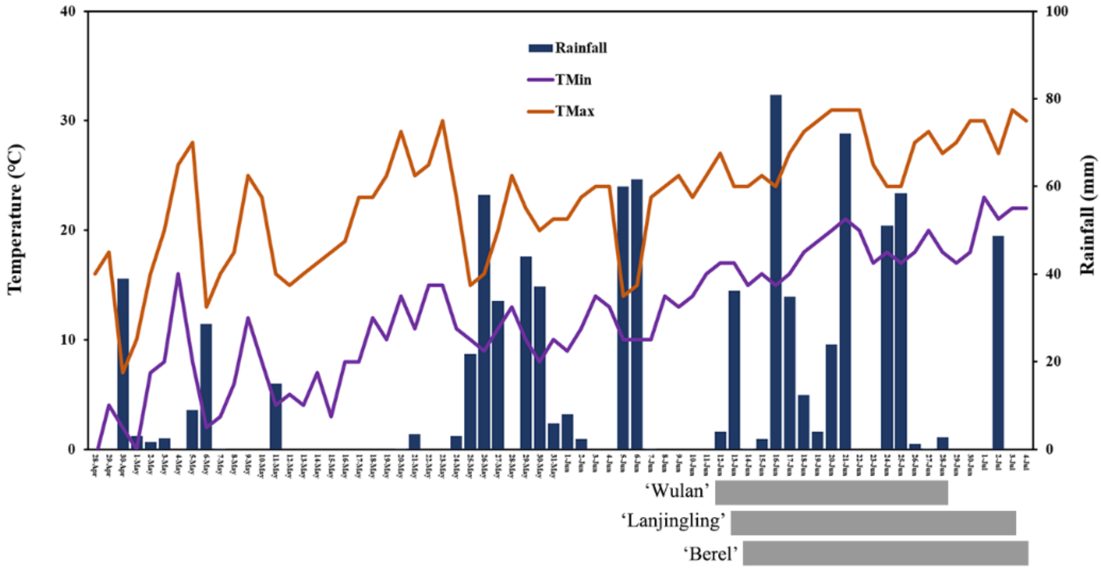

Figure S1. Maximum and minimum daily temperatures (TMax and TMin) and average daily rainfall capacity during the period from flowering to harvesting of the three blue honeysuckle cultivars in 2022

Note: Data was accessed on August 1, 2023 via https://rp5.ru/

Supplementary File S1. Extraction and determination methods for total anthocyanins, vitamin C, and phenolics

Total anthocyanins: 20μL aliquot of raw juice was extracted with 180μL of pH 4.5 buffer (0.4M sodium acetate) and pH 1.0 buffer (0.0025M potassium chloride). The extractions were performed on an orbital shaker operated at 400 rpm at room temperature for 1 h for adequate for complete extraction. The samples were then centrifuged at 8,000 rpm for 10 min. The supernatant was recovered and filtered through a 0.45 μm cellulose syringe filter before analysis, followed by measurement of absorbance values at 510 nm and 700 nm after a reaction time of 30 minutes at room temperature.

Total vitamin C: 4 g of raw juice was ground using oxalate-EDTA in a mortar, and the resulting volume was adjusted to 25 ml. Following centrifugation, 1 ml of supernatant was collected and combined with 4.0 mL of oxalate-EDTA, 1.0 mL of 5% H2SO4, 0.5 mL of metaphosphate-acetic acid, and 2.0 mL of a 5% ammonium molybdate solution. The mixture was incubated at a temperature of 30℃ for a duration of 15 minutes before measuring the absorbance at a wavelength of 705 nm.

Total phenolics: 20 μL aliquot of raw juice was vortexed with 90 μL of deionized water and 10 μL of FC reagent for 5 minutes under dark conditions. Subsequently, the mixture was further vortexed after adding 80 μL of Na2CO3. A gallic acid standard solution (0–200 mg/mL) underwent the same treatment procedure. The mixtures were left at room temperature for 2 hours before measuring the absorbance of the developed color at a wavelength of 765 nm.
